# The Physician Himself

**Published:** 1885-05

**Authors:** 


**The Physician Himself**: What he should add to his Scientific Acquirements in Order to Secure Success. By D. W. CATHELL, M. D., late Professor of Pathology in the College of Physicians and Surgeons, of Baltimore. Fourth edition. Enlarged by the addition of nearly three hundred new suggestions. Baltimore: Cushings & Bailey, 226 W. Baltimore street 1885.

We have favorably noticed the previous editions of this valuable work of Dr. Cathell. It is really an essay on "Personal Questions in Medical Practice," and contains more sound, practical sense and advice for medical men than any work we have ever read. The work is especially useful to young men just entering upon their professional life, and if some of the older members would read its pages and take some of the advice and suggestions they contain, we think they would be benefited. The work is an excellent one, and the author has performed the task well and ably. We advise our readers to obtain a copy and read it. We are sure they would gather from its contents more useful and practical hints in regard to their duties and relations to the community, and also of the measures necessary for professional success, than any work we have ever read.

